# Therapeutic Interference With Vascular Calcification—Lessons From Klotho-Hypomorphic Mice and Beyond

**DOI:** 10.3389/fendo.2018.00207

**Published:** 2018-05-04

**Authors:** Florian Lang, Christina Leibrock, Lisann Pelzl, Meinrad Gawaz, Burkert Pieske, Ioana Alesutan, Jakob Voelkl

**Affiliations:** ^1^Department of Physiology I, Eberhard Karls-University, Tübingen, Germany; ^2^Fresenius Kabi Deutschland GmbH, Bad Homburg, Germany; ^3^Department of Internal Medicine III, Eberhard Karls-University, Tübingen, Germany; ^4^Department of Internal Medicine and Cardiology, Charité-Universität Medizin Berlin, Berlin, Germany; ^5^Berlin Institute of Health (BIH), Berlin, Germany; ^6^Partner Site Berlin, German Centre for Cardiovascular Research (DZHK), Berlin, Germany

**Keywords:** vascular calcification, bicarbonate, carbonic anhydrase inhibitors, magnesium, mineralocorticoid receptor, ammonium salts, osteogenic signaling, phosphate

## Abstract

Medial vascular calcification, a major pathophysiological process associated with cardiovascular disease and mortality, involves osteo-/chondrogenic transdifferentiation of vascular smooth muscle cells (VSMCs). In chronic kidney disease (CKD), osteo-/chondrogenic transdifferentiation of VSMCs and, thus, vascular calcification is mainly driven by hyperphosphatemia, resulting from impaired elimination of phosphate by the diseased kidneys. Hyperphosphatemia with subsequent vascular calcification is a hallmark of klotho-hypomorphic mice, which are characterized by rapid development of multiple age-related disorders and early death. In those animals, hyperphosphatemia results from unrestrained formation of 1,25(OH)_2_D_3_ with subsequent retention of calcium and phosphate. Analysis of klotho-hypomorphic mice and mice with vitamin D_3_ overload uncovered several pathophysiological mechanisms participating in the orchestration of vascular calcification and several therapeutic opportunities to delay or even halt vascular calcification. The present brief review addresses the beneficial effects of bicarbonate, carbonic anhydrase inhibition, magnesium supplementation, mineralocorticoid receptor (MR) blockage, and ammonium salts. The case is made that bicarbonate is mainly effective by decreasing intestinal phosphate absorption, and that carbonic anhydrase inhibition leads to metabolic acidosis, which counteracts calcium-phosphate precipitation and VSMC transdifferentiation. Magnesium supplementation, MR blockage and ammonium salts are mainly effective by interference with osteo-/chondrogenic signaling in VSMCs. It should be pointed out that the, by far, most efficient substances are ammonium salts, which may virtually prevent vascular calcification. Future research will probably uncover further therapeutic options and, most importantly, reveal whether these observations in mice can be translated into treatment of patients suffering from vascular calcification, such as patients with CKD.

## Introduction

Medial vascular calcification is a key pathophysiological process associated with the risk of cardiovascular events in a variety of clinical conditions such as aging, diabetes, and chronic kidney disease (CKD) (1, 2). Accordingly, vascular calcification is a powerful predictor of cardiovascular and all-cause mortality (3–5). Vascular calcification in CKD results mainly from impaired renal phosphate elimination with subsequent hyperphosphatemia and precipitation of calcium-phosphate (6). Accordingly, plasma phosphate concentrations are correlated with the incidence of cardiovascular events, heart failure, and death (7, 8).

Vascular calcification results, at least in part, from an active process in vascular smooth muscle cells (VSMCs) (6). Exposure of VSMCs to enhanced extracellular phosphate concentrations is followed by osteo-/chondrogenic transdifferentiation *via* complex intracellular signaling pathways (9). Phosphate complexes with calcium to form pro-inflammatory calcium-phosphate nanoparticles (10, 11). Calcium-phosphate crystals are further involved in the formation of protein–mineral complexes, the calciprotein particles (CPPs) (12). These can transform into more toxic secondary CPPs containing crystalline calcium-phosphate (13). Osteo-/chondrogenic signaling cascades in VSMCs can be triggered by calcium-phosphate nanoparticles and/or secondary CPPs (14–20).

Osteo-/chondrogenic signaling involves upregulation of the type III sodium-dependent phosphate transporter PIT1 (also known as SLC20A1) (21, 22). The transdifferentiated VSMCs express osteogenic transcription factors, such as MSH homeobox 2 (MSX2) and core-binding factor alpha 1 (CBFA1, also known as runt-related transcription factor 2, RUNX2) as well as chondrogenic transcription factors such as SRY-Box 9 (SOX9) (23–25) to facilitate, *via* various complex mechanisms, vascular tissue mineralization (1). Vascular calcification can be prevented by inhibition of CBFA1 (26). The transcription factor NFAT5 (nuclear factor of activated T-cells 5) upregulates CBFA1 expression, an effect mediated by the transcription factor SOX9 (27). Osteo-/chondrogenic reprogramming ultimately upregulates the expression and activity of tissue non-specific alkaline phosphatase (ALPL), an enzyme hydrolyzing the calcification inhibitor pyrophosphate (28). Transdifferentiated VSMCs are also able to secrete matrix vesicles to actively promote tissue mineralization (29). Vascular osteo-/chondrogenic transdifferentiation precedes vascular calcification (30) and has been observed in vasculature of CKD patients (31). Accordingly, osteo-/chondrogenic transdifferentiation predisposes vascular tissue in CKD patients to vascular calcification (32). The orchestration of vascular calcification is, however, still incompletely understood (33).

Valuable insight into mechanisms of vascular calcification was gained by analysis of the klotho-hypomorphic mice (34). Klotho is a transmembrane protein with highest expression in kidney, but also found in parathyroid glands and choroid plexus (34). The extracellular domain of klotho is cleaved off and released into blood (35). Soluble klotho confers protection of kidneys (36) and cardiovascular system (37). Klotho counteracts tissue fibrosis (38, 39), progression of CKD (38), cardiomyopathy (38), vascular calcification (38), and tumor growth (39). Klotho is in part effective by interference with TGFβ1 signaling (39).

**Figure 1:**
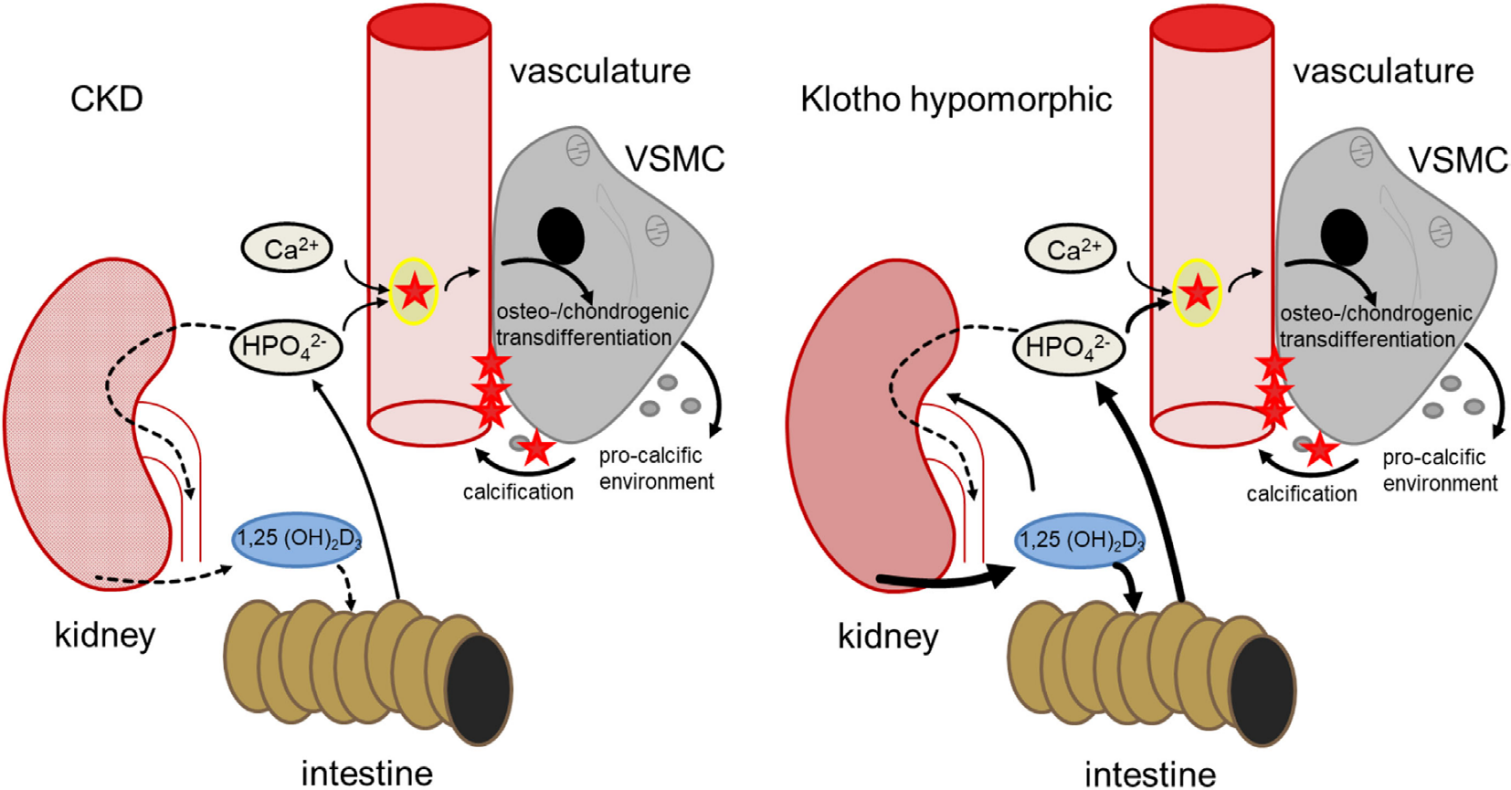
Comparison of hyperphosphatemia due to chronic kidney disease (CKD) (left) and klotho deficiency (right). In CKD, renal failure leads to impaired phosphate elimination and reduced formation of calcitriol [1,25(OH)_2_D_3_]. Phosphate overload and formation of secondary calciprotein particles (CPPs) (48) induce an osteogenic remodeling of vascular smooth muscle cells (VSMCs), causing matrix-vesicle release and a pro-calcific environment and subsequent vascular mineralization. In klotho-hypomorphic mice, klotho deficiency causes an unrestrained calcitriol formation. This causes increased intestinal and renal phosphate reabsorption, increasing phosphate levels. Therefore, despite the potential anti-calcific effects of calcitriol (49), this phosphate overload exceeds the capabilities of anti-calcific mechanisms in the body and may induce a comparable osteo-/chondrogenic transdifferentiation of VSMCs. Although the origin of hyperphosphatemia differs between CKD and kl/kl mice, both presumably suffer from excessive phosphate concentrations and increased formation of secondary CPPs to induce a similar VSMC-mediated calcification.

Klotho is required for the negative regulation of 25-hydroxyvitamin D3 1-α-hydroxylase (1-α-hydroxylase) by FGF23 and thus for inhibition of 1,25-dihydroxyvitamin D3 (1,25(OH)_2_D_3_) production (35, 40). Contrary to CKD patients, production of 1,25(OH)_2_D_3_ is excessive in klotho-hypomorphic mice, resulting in elevated phosphate levels (35). Therefore, the mice suffer from severe tissue calcification, mimicking the findings in mice with renal failure (41). These mice further display a wide variety of age-related disorders and early death (34, 35). Conversely, overexpression of klotho increases the life span of mice (42). Apparently, klotho may similarly influence the life span of humans (43). Although 1,25(OH)_2_D_3_ may exhibit protective effects during calcification (44), its excessive formation in klotho-hypomorphic mice increases intestinal calcium and phosphate uptake and renal phosphate retention, thus driving the phenotype and tissue calcification (35, 45). The life span of klotho-hypomorphic mice is substantially increased by vitamin D_3_-deficient diet (45). Moreover, klotho stimulates Na^+^/K^+^-ATPase activity (46) and lack of klotho leads to extracellular volume depletion with secondary increase of ADH and aldosterone release (40). Dehydration, in turn, downregulates klotho expression (47). Although the origin of hyperphosphatemia differs between CKD and klotho-hypomorphic mice, both lead to comparable sequelae of vascular calcification (Figure 1).

The present brief review addresses attempts to interfere with vascular calcification, premature aging and early death of klotho-hypomorphic mice and similar models. We anticipate that insights from maneuvers successful in klotho-hypomorphic mice may improve our understanding of the mechanisms underlying calcifications in patients with CKD.

## Bicarbonate

Most CKD patients and klotho-hypomorphic mice suffer from acidosis (50, 51), which may further enhance plasma phosphate concentrations (52) and aggravate CKD (53–57). Conversely, alkali administration may slow the progression of CKD (53–56). In contrast to rats, in which metabolic acidosis has been shown to slow the progression of renal disease (58–60), in CKD patients, the deterioration of renal function is accelerated by acidosis and slowed by bicarbonate treatment (56, 61, 62).

Bicarbonate treatment of klotho-hypomorphic mice decreased tissue calcification and increased the average life span of those mice (63). Bicarbonate treatment did not significantly modify plasma concentrations of 1,25(OH)_2_D_3_ and calcium, but significantly decreased plasma phosphate concentrations and plasma aldosterone concentrations (63). Bicarbonate treatment was presumably primarily effective by decreasing intestinal phosphate absorption and renal phosphate reabsorption (63). Alkalinization of the intestinal lumen is expected to compromise phosphate solubility and absorption.

## Carbonic Anhydrase Inhibition

Extracellular pH can be modified by treatment with carbonic anhydrase inhibitors, such as acetazolamide (64). The diuretic interferes with proximal tubular bicarbonate reabsorption and, thus, leads to renal bicarbonate loss and acidosis (64). Extracellular pH has a profound effect on calcium and phosphate solubility, which is enhanced by acidification and decreased by alkalinization (65). Moreover, acidosis counteracts vascular calcification by downregulation of PIT1 expression (58, 65, 66) and inhibition of renal tubular phosphate reabsorption with increase of renal phosphate elimination (67).

Acetazolamide treatment of klotho-hypomorphic mice blunted the calcifications in trachea, lung, kidney, stomach, intestine, and vascular tissues, reversed the excessive aortic *Alpl* transcript levels as a marker of aortic osteo-/chondrogenic signaling, increased the plasma concentrations of the calcification counteracting proteins osteoprotegerin, osteopontin as well as fetuin-A (68–70) and, thus, tripled the life span despite unaltered plasma concentrations of FGF23, 1,25(OH)_2_D_3_, calcium and phosphate (64). *In vitro*, acidic medium prevented the phosphate-induced upregulation of *ALPL* mRNA expression in primary human aortic smooth muscle cells, indicating that extracellular acidosis interferes with osteo-/chondrogenic transdifferentiation of VSMCs (64). Acidic conditions may impair the formation of small calcium-phosphate complexes during hyperphosphatemia and, thus, hinder VSMC osteo-/chondrogenic transdifferentiation.

It should be kept in mind that the bicarbonaturia and, thus, systemic acidosis following carboanhydrase inhibitor treatment depends on renal function and may, thus, be lacking in CKD patients.

## Magnesium

In CKD patients, lower serum magnesium levels are associated with vascular calcification (71) and are predictive for increased arterial stiffness and mortality (72). Previous *in vitro* studies have shown that magnesium treatment is able inhibit phosphate-induced VSMCs calcification (73–75). Magnesium is able to interfere with hydroxyapatite formation (76). Also, magnesium interferes with osteo-/chondrogenic reprogramming of VSMCs.

Experiments in mice treated with excessive levels of vitamin D_3_, mimicking excessive vitamin D receptor activation during klotho deficiency, revealed magnesium supplementation as a further potential treatment to reduce the progression of vascular calcification (77). Vitamin D_3_ overload was followed by extensive vascular calcification and upregulation of aortic osteoinduction as shown by expression of the osteogenic markers *Msx2, Cbfa1*, and *Alpl* (77). Those effects were blunted by additional treatment with MgCl_2_. Vitamin D_3_ overload upregulated the aortic expression of calcium-sensing receptor (CASR), an effect augmented by additional MgCl_2_ supplementation (77). Magnesium can activate CASR (78) and CASR activation in VSMCs inhibits osteo-/chondrogenic remodeling and calcification (79).

Those *in vivo* observations were supported by *in vitro* experiments using primary human aortic VSMCs. Addition of MgCl_2_ to the VSMCs cell culture medium reversed the phosphate-induced calcification and osteo-/chondrogenic signaling, effects paralleled by upregulation of CASR expression. The protective effects of MgCl_2_ were virtually abrogated by the CASR antagonist NPS-2143 or by silencing of the CASR gene (77). Thus, magnesium supplementation may reduce the progression of vascular calcification at least in part by activating CASR. Magnesium supplementation may thus be beneficial in CKD patients (80). Recently, a first pilot trial indicated that magnesium supplementation is safe in CKD patients and is able to reduce serum calcification propensity (81).

## Mineralocorticoid Receptor (MR) Inhibition

Vascular smooth muscle cells express the MR (82) and MR stimulation by aldosterone triggers the osteo-/chondrogenic signaling (82–87) by upregulation of PIT1 expression (87, 88), leading to expression of osteogenic transcription factors and enzymes and subsequent mineralization (87). Klotho-hypomorphic mice develop renal sodium loss and hyperaldosteronism (89). Hyperaldosteronism presumably contributes to the stimulation of vascular calcification in klotho-hypomorphic mice (40, 87, 90) and CKD patients (91). Accordingly, treatment with the MR antagonist spironolactone reduces the extent of vascular calcification in klotho-hypomorphic mice and rats with adenine-induced renal failure (88) and reduces cardio-/cerebrovascular mortality in dialysis patients (92). Spironolactone treatment of klotho-hypomorphic mice reduced aortic PIT1-dependent osteoinductive signaling, but increased cystatin-C levels (87). MR blockade with spironolactone may particularly suppress the progression of vascular calcification in patients with hyperaldosteronism.

Spironolactone may be effective even at normal levels of circulating aldosterone (93, 94). Aldosterone is produced not only in adrenal glands, but in diverse tissues (95–98) including the vasculature (99). Aldosterone synthase (also known as CYP11B2) is expressed during calcifying conditions and, thus, aldosterone may be produced in VSMCs (99, 100). Vascular aldosterone production is particularly important under pathological conditions (97). Vascular aldosterone may foster development of hypertension (101). CYP11B2 is upregulated in atheroma-plaques (102) and contributes to oxidative stress (103). In accordance, high-phosphate treatment increased aldosterone synthase expression in VSMCs (90) and silencing of aldosterone synthase attenuated the phosphate-induced osteo-/chondrogenic transdifferentiation and calcification *in vitro*. Similarly, aldosterone synthase expression is higher in coronary arteries from patients with impaired renal function and correlated with *CBFA1* expression. Aldosterone synthase expression in VSMCs is upregulated by disruption of APEX1-dependent gene suppression (90). Accordingly, APEX1 is protective against VSMC calcification (90, 104).

Aldosterone synthase expression is similarly enhanced in klotho-hypomorphic mice (90). In those mice, aortic osteo-/chondrogenic signaling is decreased by spironolactone, but not by adrenalectomy and in adrenalectomized klotho-hypomorphic mice, spironolactone treatment still significantly blunts aortic osteoinductive reprogramming (90).

Mineralocorticoid receptor antagonism may, thus, be a therapeutic option for hyperphosphatemic patients even in the absence of hyperaldosteronism (86). Spironolactone may further protect VSMCs in diabetes (105), which may lead to upregulation of vascular aldosterone synthase (100).

The effects of spironolactone in CKD patients are under study (106, 107). Clinical trials indicate that spironolactone treatment reduces morbidity and mortality in hemodialysis patients (92). MR inhibition may cause a transient reduction of renal function and promote hyperkalemia, but has been shown to be relatively safe in CKD patients (92, 108).

## Ammonium Salts

Besides its acidifying effect on extracellular pH (109, 110), ${\text{NH}}_{4}^{+}$ may dissociate to H^+^ and NH_3_ which easily crosses membranes, thus entering cells and cellular compartments (111). In acidic intracellular compartments NH_3_ binds H^+^ and is trapped as ${\text{NH}}_{4}^{+}$ (112). The binding of H^+^ alkalinizes acidic cellular compartments (113) and the intracellular/intra-compartimental accumulation of ${\text{NH}}_{4}^{+}$ swells cells and acidic intracellular compartments (114–116). Cell swelling may downregulate the cell volume sensitive transcription factor NFAT5 (117, 118). Moreover, alkalinization of acidic cellular compartments may interfere with the maturation of several proteins including TGFβ1 (119), a key factor in the regulation of osteo-/chondrogenic signaling of VSMCs (120–122).

Treatment of klotho-hypomorphic mice with NH_4_Cl containing drinking water prevented soft tissue and vascular calcifications and increased their life span more than 12- (♂) or 4-fold (♀) without significantly affecting extracellular pH or plasma concentrations of 1,25(OH)_2_D_3_, calcium, and phosphate (123). Tissue calcification and aging were further delayed in klotho-hypomorphic mice by NH_4_NO_3_ (124).

NH_4_Cl prevents vascular calcification apparently not by inducing acidosis. Untreated klotho-hypomorphic mice suffer from respiratory acidosis resulting from severe lung emphysema (123). NH_4_Cl treatment prevents the development of lung emphysema and, thus, respiratory acidosis (123). Instead, NH_4_Cl induces a metabolic acidosis of similar extracellular pH as in untreated mice (123).

NH_4_Cl treatment prevents development of extracellular volume depletion, thus normalizing ADH release and plasma aldosterone levels (40). The decrease of plasma aldosterone concentrations following NH_4_Cl treatment presumably contributes to the decrease of vascular calcification. However, the effect of NH_4_Cl on survival and calcification (123) is, by far, larger than that of aldosterone receptor blockade (87).

NH_4_Cl treatment is presumably mainly effective by interference with osteo-/chondrogenic transdifferentiation of VSMCs (123). In aortic tissue of klotho-hypomorphic mice and in phosphate treated VSMCs *in vitro*, NH_4_Cl disrupted the increased expression of osteogenic and chondrogenic markers *CBFA1* and *SOX9* and of ALPL (123). Osteo-/chondrogenic reprogramming is paralleled by VSMCs senescence (125), and thus vascular aging (126). NH_4_Cl treatment reversed the upregulation of *PAI-1, p21*, and *GLB1*, key elements in the orchestration of senescence (127). *TGFβ1* expression was upregulated in aortic tissue of klotho-hypomorphic mice and in phosphate treated VSMCs, which in turn triggers cellular senescence, osteo-/chondrogenic reprogramming and aging (128) and is decreased by NH_4_Cl treatment (123). NH_4_Cl further impairs maturation of TGFβ1 (119). TGFβ1 is a stimulator of NFAT5 expression (129). NH_4_Cl treatment reverses the enhanced expression of *NFAT5* in klotho-hypomorphic mice and in phosphate treated VSMCs (123). Addition of exogenous TGFβ1 protein or NFAT5 overexpression triggers osteo-/chondrogenic reprogramming of VSMCs *in vitro*, which cannot be reversed by NH_4_Cl, indicating that NH_4_Cl is effective upstream of mature TGFβ1 (123). NH_4_Cl treatment is, at least in part, effective by disrupting TGFβ1-dependent osteo-/chondrogenic signaling (123) and, thus, vascular calcification (130).

Further *in vitro* experiments revealed that inhibition of the vacuolar H^+^ ATPase with bafilomycin A1 or following dissipation of the pH gradient across the membranes of acidic cellular compartments with methylamine similarly disrupted phosphate-induced TGFβ1-dependent osteo-/chondrogenic signaling in VSMCs (131), supporting the hypothesis that vascular acidic cellular compartments are necessary for promoting vascular calcification.

A concern of NH_4_Cl treatment is the cerebral ammonia toxicity (114–116). However, the employed NH_4_Cl dosage does apparently not lead to toxic ammonia concentrations as NH_4_Cl treated male klotho-hypomorphic mice reached a life span close to that of untreated wild-type mice, and behavioral studies did not reveal any defect in NH_4_Cl treated wild-type mice (123). Needless to say, NH_4_Cl treatment may be hazardous in patients with hepatic failure.

## Conclusion

**Figure 2:**
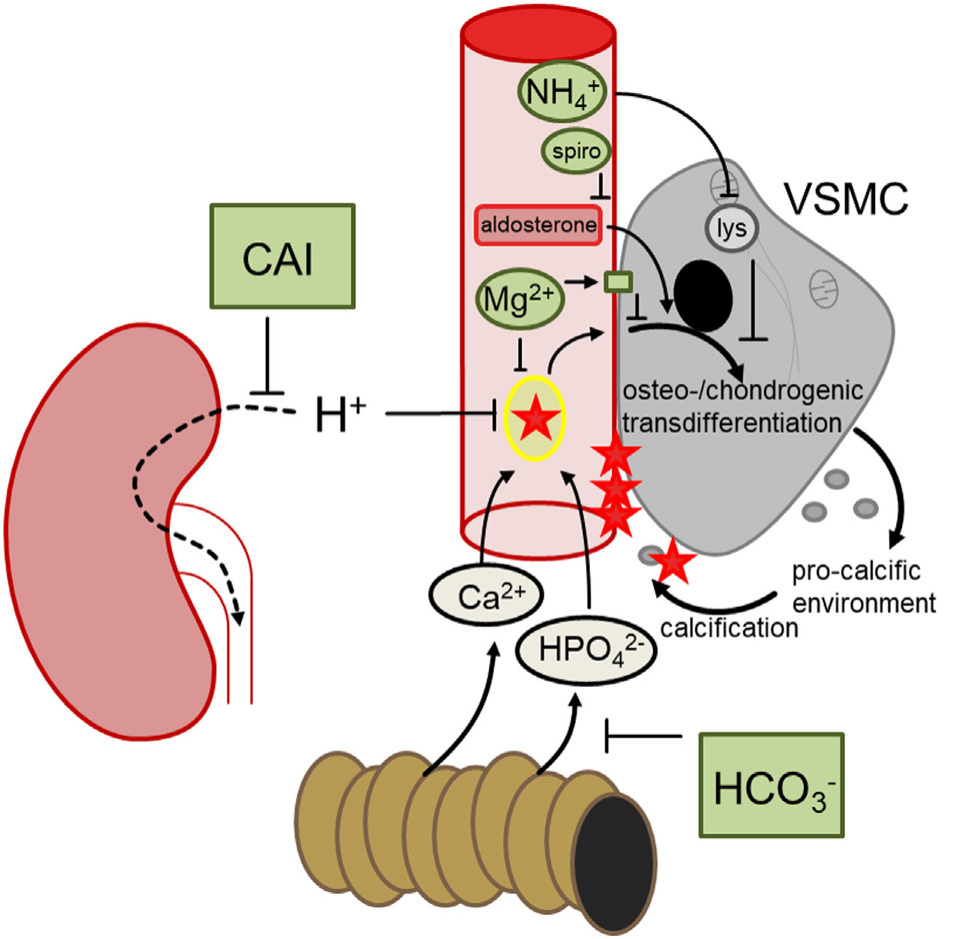
Hypothetical mechanisms involved in the discussed treatments preventing vascular calcification. During hyperphosphatemia, phosphate complexes with calcium and forms secondary calciprotein particles, inducing osteo-/chondrogenic transdifferentiation of vascular smooth muscle cells (VSMCs), which in turn generates a pro-calcific environment and subsequent active tissue mineralization. Oral bicarbonate treatment may impair intestinal phosphate reabsorption, ameliorating hyperhosphatemia. Renal carboanhydrase inhibition causes proton retention, which may reduce the formation of calcium-phosphate nanoparticles. Magnesium may similarly directly prevent calcium-phosphate complexation and stimulates the calcium sensing receptor in VSMCs, blunting osteo-chondrogenic transdifferentiation. This transdifferentiation is also directly stimulated by aldosterone, which may be blunted by spironolactone (spiro). Ammoniumchloride leads to lysosomal (lys) alkalinization which appears to dissipate the osteo-chondrogenic transdifferentiation of VSMCs. These mechanisms may provide the basis to develop a therapeutic approach to reduce the burden of vascular calcification.

Klotho-hypomorphic mice and mice with vitamin D_3_ overload suffer from severe vascular calcification. Experiments in those animals shed novel light on the mechanisms orchestrating vascular calcification and led to the discovery of several powerful therapeutic opportunities (Figure 2). The most effective treatment turned out to be NH_4_Cl, but also acetazolamide, spironolactone, bicarbonate, or magnesium supplementation were able to reduce the progression of vascular calcification *in vivo*. Further studies are required to fully define advantages and disadvantages of those treatments, to possibly uncover additional therapeutic options and—most importantly—to clarify whether the successful treatments in mice can be translated into avoidance of vascular calcification in human disease, such as CKD, diabetes, and (premature) aging.

## Author Contributions

All authors listed have made substantial, direct and intellectual contribution to the work, and approved it for publication.

## Conflict of Interest Statement

CL is employed by the company Fresenius Kabi Deutschland GmbH, Bad Homburg, Germany. All other authors declare no competing interests.
